# Supplementary material for: Chromothripsis during telomere crisis is independent of NHEJ, and consistent with a replicative origin
Source: Genome Res. 2019 May;29(5):737–49. doi: 10.1101/gr.240705.118 (PMC6499312; doi:10.1101/gr.240705.118)
Supplement: Supplemental Material [file supp_gr.240705.118_Supplemental_file_1.zip › contigs/annotated_contigs/DB46/contig.2.DB46_length_866_mean_cov_7.62124711316.docx]

**DB46_length_866_mean_cov_7.62124711316**

```
TCTATTAATCTTTTCTATTATAGCTTCTGGGTTTTGTGTGATGTTTGGAAAGGCTCTCTCTACTTCTTTGTGGTTAAAGTTCTTTAATT
 >chr12:65279662-65280101 + E=3e-252
TTTTCCTTCTACTAGTTTTATGGTTTCATTTTTTATGTTTAAATCTGACTCATTTGGAATTTACTTTTGTGTAAGTTGGGAACTAAGGT

TCCATCTTAACATTTATTTTTTCCAAATAGTTACCTAGTGGTGCCGGCACCATTTATTAATCTTTCCCCTATTGATTAAAAATGCCACC

TTTATAATCACATACTAAATCCCTGTATGTTTTAGGGTGAATATCTGGACTTTTGGAGTACAACTGAACCACTACCCTCATTCAGGTGG

GGGCAGAGAGGCCAGAGCTCTGCACACTGGTTTCCTCTCTGGTTGCCAGCATTACCCCTGAAGTTGCTTTGGGGGAGATC|TTG|TTTA
                                                                                 >chr12
AAGGCTTGCCAAAAACACAAAACAAAGCCTTAAACCCCAAACCTTTAATAGTATAAAAATAACAAGGCTTAAGTCATAAACATTGGATT
:65281152-65281582 + E=1e-234
TCCAGAATTTAAAAAGATAATTTTTGAAAAAGGAATTACATCAAGAAAATTTTTTTTGAATTGACTGTCTTCTAAAACAAATTAAAAAA

ACAAAAACAAGAAAAGCAATTAAAAAAAACAGTTGCTAATGTTCAATCCGGAGAAATGGATGGATTCCAGTATTTTATTCACAAACAAG

CTTCATCCAAATGGCAGAACAACAGGGGTCTGGCTTCTGCATGGCTCAGGCCTGAGTTTCTGAACCGGAGGGTATATGGTGAGGTTGGG

ATTAAGGGTTTTCCTGCCTCATTTGGGTAGGGCCTAGTCACAGTTGCACCAGCAGTTGTGTTCACAT
```
